# Synthesis and Antifungal Evaluation of Novel *N*-Alkyl Tetra- and Perhydroquinoline Derivatives

**DOI:** 10.3797/scipharm.1409-13

**Published:** 2014-11-22

**Authors:** Jürgen Krauß, Michal Hornacek, Christoph MÜller, Verena Staudacher, Martina Stadler, Franz Bracher

**Affiliations:** Department of Pharmacy – Center for Drug Research, Ludwig-Maximilian-University, Butenandtstr. 5–13, 81377 Munich, Germany

**Keywords:** Quinoline, Antifungal activity, Ergosterol biosynthesis, Δ8,7-Isomerase, Enzyme inhibitor

## Abstract

A series of novel *N*-alkyl tetra- and perhydroquinoline derivatives and their hydrochlorides were prepared from tetrahydro- or *trans*-perhydroquinoline by direct alkylation with alkyl halides and subsequent precipitation with HCl gas. The antimicrobial activity of the resulting amines was evaluated in an agar diffusion assay. The minimal inhibitory concentrations (MIC) of the active compounds were determined by the microdilution method. In contrast to the tetrahydroquinolines, the perhydro analogues showed significant antifungal activity. In an assay for the detection of target enzymes in ergosterol biosynthesis, *N*-undecylperhydroquinoline was identified as an inhibitor of Δ8,7-isomerase.

## Introduction

**Fig. 1:**
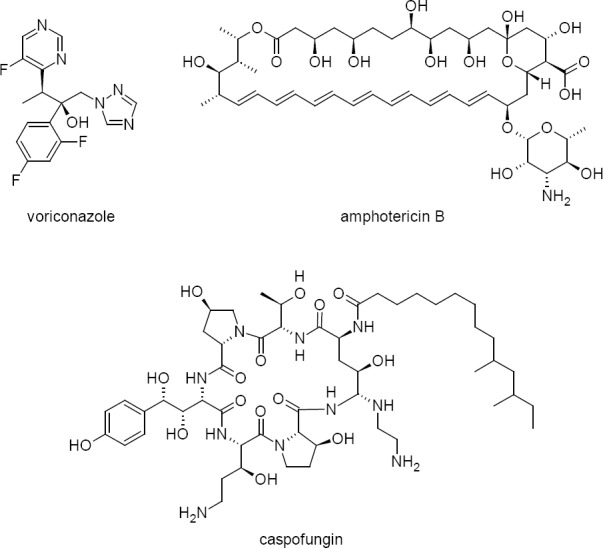
Drugs used for the treatment of systemic fungal infections

In the last few decades, a dramatic increase in fungal infections was observed in the Northern Hemisphere. Especially cancer patients, organ-engrafted patients, and immunecompromised patients (e.g. AIDS patients) are predisposed to systemic fungal infections by *Candida* or *Aspergillus* species with a high lethality. Only a few drugs from three classes can be used in the treatment of these life-threatening systemic infections: azoles (e.g. fluconazole, posaconazole, or voriconazole), polyene macrolides (e.g. amphotericin B), and echinocandins (e.g. caspofungin, anidulafungin, or micafungin) [1, 2] (Fig. 1).

Ergosterol biosynthesis is an extremely important target in the development of new antimycotic drugs [3]. Ergosterol and the enzymes in the post-squalene part of ergosterol biosynthesis are specific for fungi, so specific inhibitors should be selective for the fungal pathway. By now, only the four enzymes, squalene epoxidase (by allylamines), C14 demethylase (by azoles), and Δ8,7-isomerase and Δ14-reductase (both by morpholines), of this complex pathway are targeted by antimycotics used in human medicine.

**Fig. 3:**
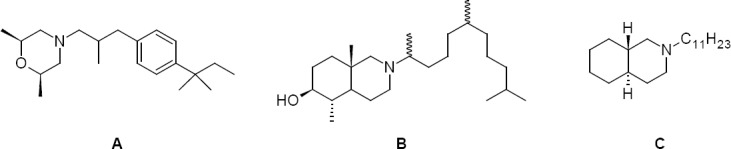
Potent Δ14-reductase and Δ8,7-isomerase inhibitors.

The mimicry of carbocationic high energy intermediates of this biosynthesis pathway by protonated amines is an often-used approach towards inhibitors of Δ8,7-isomerase and Δ14-reductase. The most important drug in human medicine using this concept is the morpholine amorolfine (**A**), but this drug can only be used in topical formulations [4] (Fig. 3). Several morpholine antifungals like fenpropimorph or tridemorph, showing the same mechanism of action, are used in agrochemistry.

The same mechanism of action could be shown for *N*-alkylpiperidines like fenpropidin [5–7]. Rahier and coworkers found the same mechanism of action for complex *N-*alkyl-perhydroisoquinolines (**B**). In our group, *N*-n-undecyl-*trans*-decahydroisoquinoline (**C**) was identified as a potent antifungal inhibiting the enzyme Δ8,7-isomerase [8] (Fig. 3). Related imidazol-5-yl carbinols showed comparable antifungal activities, but surprisingly did not interfere with ergosterol biosynthesis [9].

The surprisingly high antifungal activities of simple *N*-alkyldecahydroisoquinolines and imidazol-5-yl carbinols, the observed outstanding role of the length of the alkyl side chain in both series, and the surprising differences in molecular mechanisms of actions prompted us to perform analogous investigations on a third heterocyclic scaffold, the quinoline ring system.

## Results and Discussion

In continuation of our above-mentioned work [8, 9], we evaluated the antifungal potency of simple *N*-alkyl tetrahydro- and perhydroquinoline derivatives in the present work. As we found in previous work that *N*-n-alkyl substitutents with nine to twelve carbons led to the highest antifungal potency, we focused on side chains with a length of C_9_ to C_12_.

In a first series, 1,2,3,4-tetrahydroquinoline (**1a**), first deprotonated with NaH, was alkylated with unbranched C_9_ to C_12_ alkyl halides to give the tertiary amines **2a–d**. The amines were dissolved in dry diethyl ether and precipitated with HCl gas to give the more stable hydrochlorides **3a–d**. In an alternative approach, **2d** was prepared from **1a** and dodecanoyl chloride to give the amide **6**, which was reduced with LiAlH_4_ to give **2d** (Scheme 1).

**Sch. 1:**
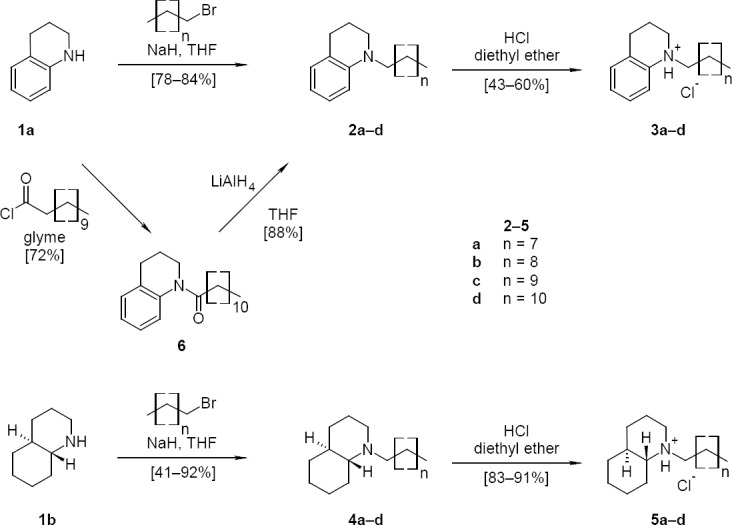
Synthesis of *N*-alkyl tetrahydro- and decahydroquinoline derivatives

In a second series, (±)-*trans-*perhydroquinoline (**1b**) was alkylated with C_9_ to C_12_ alkyl halides in the same way as described above to give the tertiary amines **4a–d**, which were precipitated with HCl gas to give the hydrochlorides **5a–d** (Scheme 1). The *trans* stereochemistry was selected since it resembles the stereochemistry typically found in the connections of the rings in ergosterol intermediates. The *trans* configuration of **1b** was confirmed by comparison of ^13^C-NMR data with literature data [10].

The resulting compounds and known compounds, 1-undecylpiperidine (**7**) [14] and 1-decylpiperazine (**8a**) [15], as well as 1-methyl-4-undecylpiperazine (**8b**) [16] (prepared in the same way), were tested in an agar diffusion assay [11] against Gram-positive (*Staphylococcus equorum* (DSMZ-Nr. 20675), *Streptococcus entericus* (DSZM-Nr. 14446)) and Gram-negative bacteria (*Escherichia coli* (DSMZ-Nr. 426), *Pseudomonas marginalis* (DSMZ-Nr. 7527)), as well as the fungi *Yarrowia lipolytica* (DSMZ-Nr. 1345), *Candida glabrata* (DSMZ-Nr.11226), *Aspergillus niger* (DSMZ-Nr. 1988), and *Hyphopichia burtonii* (DSMZ-Nr. 70663) (Table 1).

**Tab. 1:**
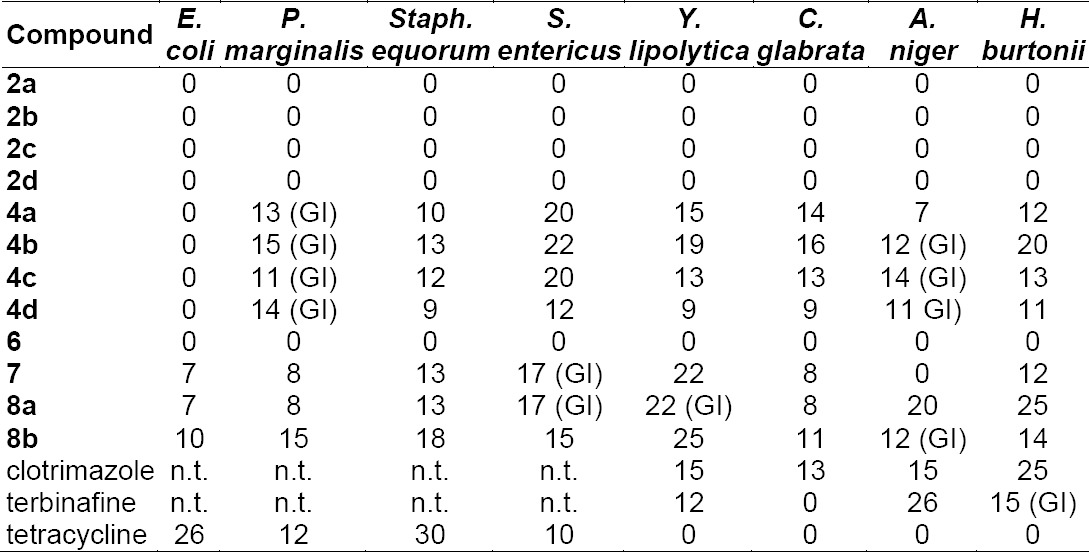
Results of the agar diffusion assay [50 µg/disc, diameter of zones of total inhibition [mm], GI = growth inhibition]

The minimal inhibitory concentrations (MIC) of the most active compounds from the agar diffusion assay were determined in a microdilution assay on *Candida glabrata, Yarrowia lipolytica*, and *Saccharomyces cerevisiae* [11]. For comparison, the *N*-undecylperhydroisoquinoline (**C**) [8] was also tested here. For solubility reasons, the hydrochlorides were used in this assay (Table 2).

**Tab. 2:**
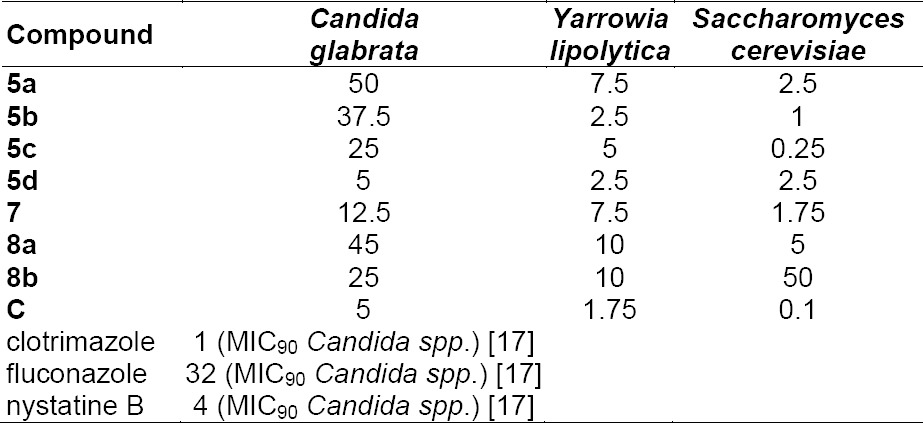
Minimal inhibitory concentrations (MIC [µg/mL] values are the arithmetic average of two determinations)

The cytotoxicity of the compounds **3a–d**, **5a–d**, and **8b** was determined in an MTT test [13] against a human leukemia cell line (HL 60). All compounds tested showed a moderate cytotoxicity against this cell line with IC_50_ values between 6 to 46 µM (Table 3).

**Tab. 3:**
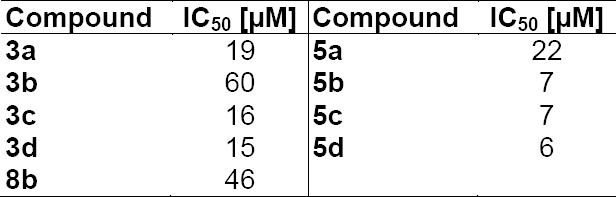
IC_50_ values against a HL 60 cell line (IC_50_ values are the arithmetic average of three determinations)

The compounds **5c** and **7** were also subjected to our whole-cell assay for identification of the target enzyme in ergosterol biosynthesis [12]. In this assay, the strains *Candida glabrata* and *Saccharomyces cerevisiae* were incubated with the test compounds, and after cell lysis, the changes in the sterol pattern were analyzed by GLC-MS. The accumulation of the Δ8(9)-sterol lichesterol (ergosta-5,8,22-trien-3β-ol) clearly indicates an inhibition of the enzyme Δ8,7-isomerase. Both compounds **5c** and **7** showed an accumulation of lichesterol, so one mechanism of action is an inhibition of Δ8,7-isomerase.

## Conclusion

The *N*-alkyl tetrahydroquinoline compounds **2a–d** showed no antibiotic or antimycotic activity against the tested microorganisms. Their corresponding hydrochlorides **3a–d** showed weak cytotoxicity. In contrast, the (±)-*trans*-*N*-alkylperhydroquinolines showed high antimycotic activity comparable to the commonly used drug clotrimazole. The maximum of activity was found with the C_10_ alkyl chain in the agar diffusion assay and with the C_12_ alkyl chain in the MIC determination, shorter alkyl chains led to a decrease in activity, as already found for other *N*-alkyl heterocycles [8, 9]. Compared to the recently described [8] *N*-alkyl perhydroisoquinolines (e.g. **C**), the new perhydroquinoline compounds showed similar antifungal activity, but higher cytotoxicity against a human cell line. Both perhydroquinolines and perhydroisoquinolines target the same enzyme in ergosterol biosynthesis (Δ8,7-isomerase), but the latter chemotype seems to have benefits in selectivity.

## Experimental

### General

IR-spectra: Perkin-Elmer FT-IR Paragon 1000; MS: Hewlett Packard MS-Engine; electron ionisation (EI) 70 eV, chemical ionisation (CI) with CH_4_ (300 eV); NMR: JNM-Eclipse+400 (400 MHz) (^1^H: 400 MHz, ^13^C: 100 MHz), and Avance III HD 400 MHz Bruker BioSpin (400 MHz); melting points: Büchi Melting Point B-540 (not corrected); flash column chromatography (FCC): silica gel 60 (230–400 mesh, E. Merck, Darmstadt); GLC-MS: Shimadzu GC-17 A (carrier: He, oven temperature program: 100–280°C, 10°C / min, capillary column: Varian VF-5 ms 30 m × 0.25, split injector T = 250°C, detector T = 260°C).

### General Procedure I (N-alkylation)

The quinoline derivative (about 4 mmol) was dissolved in 50 mL dry THF and 3 equiv. of NaH were added. The suspension was refluxed for 1 h. Then 1.4 to 2 equiv. of the alkyl halide in 5 mL dry THF was added and the mixture was refluxed for 6 h. The mixture was quenched with 50 mL 10% aqueous NaOH and was extracted with ethyl acetate (3 × 50 mL). The combined organic layers were dried over Na_2_SO_4_, the solvent was evaporated, and the residue was purified by flash column chromatography.

### General Procedure II (Preparation of Hydrochlorides)

1.0 mmol of the *N*-alkyl quinoline derivative was dissolved in 30 mL of dry diethyl ether and the solution was flushed with HCl gas for five minutes. The solvent was evaporated and the residue was disperged in 30 mL of dry diethyl ether, the suspension was placed in a fridge for 3 h and the precipitate was separated to give the analytically pure hydrochlorides.

### 1-Nonyl-1,2,3,4-tetrahydroquinoline (2a)

The compound was prepared according to General Procedure I from 0.535 g (4.01 mmol) 1,2,3,4-tetrahydroquinoline and 1.56 g (7.51 mmol) 1-bromononane to give 0.81 g (78%) as a pale yellow oil. ^1^H-NMR (400 MHz, CDCl_3_, TMS): δ 0.88 (t, *J* = 7.2 Hz, 3 H, CH_3_), 1.20–1.37 (m, 12 H, 6 CH_2_), 1.57 (m, 2 H, CH_2_), 1.93 (tt, *J* = 5.7 Hz, *J* = 6.4 Hz, 2 H, CH_2_, 3-H), 2.74 (t, *J* = 6.4 Hz, 2 H, CH_2_, 4-H), 3.21 (t, *J* = 7.6 Hz, 2 H, CH_2_), 3.27 (t, *J* = 5.7 Hz, 2 H, CH_2_, 2-H), 6.53 (dd, *J* = 7.3 Hz, *J* = 7.4 Hz, 1 H, 6-H), 6.55 (d, *J* = 8.2 Hz, 1 H, 8-H), 6.92 (d, *J* = 7.3 Hz, 1 H, 5-H), 7.03 (dd, *J* = 7.4 Hz, *J* = 8.2 Hz, 1 H, 7-H). ^13^C-NMR (125 MHz, CDCl_3_, TMS): δ 14.1 (CH_3_), 22.2 (CH_2_), 22.7 (CH_2_), 26.1 (CH_2_), 27.3 (CH_2_), 28.2 (CH_2_), 29.3 (CH_2_), 29.6 (CH_2_), 29.6 (CH_2_), 31.89 (CH_2_), 49.4 (CH_2_), 51.5 (CH_2_), 110.4 (arom. CH), 115.1 (arom. CH), 122.1 (quat. C), 127.0 (arom. CH), 129.1 (arom. CH), 145.3 (quat. C). IR (KBr), ν, cm^−1^: 3409, 3065, 3017, 2926, 2854, 1655, 1602, 1505, 1457, 1346, 1215, 1195, 1108, 1059, 743. MS (CI, m/z, %): 260 ([M+1]^+^, 100), 146 (28). MS (EI, m/z, %): 259 ([M]^+^, 12), 146 (100). HRMS: Calcd. for C_18_H_29_N: 259.2300. Found: 259.2293.

### 1-Decyl-1,2,3,4-tetrahydroquinoline (2b)

The compound was prepared according to General Procedure I from 0.525 g (3.94 mmol) 1,2,3,4-tetrahydroquinoline and 1.659 g (7.50 mmol) 1-bromodecane to give 0.91 g (84%) of **2b** as a pale yellow oil. ^1^H-NMR (400 MHz, CDCl_3_, TMS): δ 0.88 (t, *J* = 7.2 Hz, 3 H, CH_3_), 1.26–1.31 (m, 14 H, 7 CH_2_), 1.57 (m, 2 H, CH_2_), 1.94 (tt, *J* = 5.7 Hz, *J* = 6.4 Hz, 2 H, CH_2_, 3-H), 2.74 (t, *J* = 6.4 Hz, 2 H, CH_2_, 4-H), 3.21 (t, *J* = 7.6 Hz, 2 H, CH_2_), 3.27 (t, *J* = 5.7 Hz, 2 H, CH_2_, 2-H), 6.52 (dd, *J* = 7.3 Hz, *J* = 7.6 Hz, 1 H, 6-H), 6.56 (d, *J* = 8.1 Hz, 1 H, 8-H), 6.92 (d, *J* = 7.3 Hz, 1 H, 5-H), 7.02 (dd, *J* = 7.6 Hz, *J* = 8.1 Hz, 1 H, 7-H). ^13^C-NMR (100 MHz, CDCl_3_, TMS): δ 14.1 (CH_3_), 22.2 (CH_2_), 22.7 (CH_2_), 26.1 (CH_2_), 27.3 (CH_2_), 28.2 (CH_2_), 29.3 (CH_2_), 29.6 (CH_2_), 29.6 (CH_2_), 29.7 (CH_2_), 31.9 (CH_2_), 49.4 (CH_2_), 51.5 (CH_2_), 110.4 (arom. CH), 115.1 (arom. CH), 122.1 (quat. C), 127.0 (arom. CH), 129.1 (arom. CH), 145.3 (quat. C). IR (KBr), ν, cm^−1^: 3408, 3065, 3019, 2925, 2853, 1661, 1602, 1505, 1457, 1346, 1212, 1196, 1108, 1059, 742. MS (CI, m/z, %): 274 (M^+^+1, 100), 146 (28). MS (EI, m/z, %): 273 (M^+^, 10), 146 (100). HRMS: Calcd. for C_19_H_31_N: 273.2456. Found: 273.2457.

### 1-Undecyl-1,2,3,4-tetrahydroquinoline (2c)

The compound was prepared according to General Procedure I from 0.504 g (3.78 mmol) 1,2,3,4-tetrahydroquinoline and 1.743 g (7.41 mmol) 1-bromoundecane to give 0.88 g (81%) of **2c** as a pale yellow oil. ^1^H-NMR (400 MHz, CDCl_3_, TMS): δ 0.88 (t, *J* = 7.2 Hz, 3 H, CH_3_), 1.26–1.31 (m, 16 H, 8 CH_2_), 1.57 (m, 2 H, CH_2_), 1.93 (tt, *J* = 5.7 Hz, *J* = 6.3 Hz, 2 H, CH_2_), 2.74 (t, *J* = 6.3 Hz, 2 H, CH_2_, 4-H), 3.21 (t, *J* = 7.6 Hz, 2 H, CH_2_), 3.26 (t, *J* = 5.7 Hz, 2 H, CH_2_, 2-H), 6.53 (dd, *J* = 7.1 Hz, *J* = 7.3 Hz, 1 H, 6-H), 6.56 (d, *J* = 7.5 Hz, 1 H, 8-H), 6.93 (d, *J* = 7.1 Hz, 1 H, 5-H), 7.03 (dd, *J* = 7.3 Hz, *J* = 7.5 Hz, 1 H, 7-H). ^13^C-NMR (100 MHz, CDCl_3_, TMS): δ 14.1 (CH_3_), 22.2 (CH_2_), 22.7 (CH_2_), 26.1 (CH_2_), 27.3 (CH_2_), 28.2 (CH_2_), 29.4 (CH_2_), 29.6 (CH_2_), 29.6 (CH_2_), 29.6 (CH_2_), 29.7 (CH_2_), 31.9 (CH_2_), 49.4 (CH_2_), 51.5 (CH_2_), 110.4 (arom. CH), 115.1 (arom. CH), 122.0 (quat. C), 127.0 (arom. CH), 129.1 (arom. CH), 145.3 (quat. C). IR (KBr), ν, cm^−1^: 3407, 3064, 3019, 2925, 2853, 1656, 1602, 1505, 1457, 1346, 1195, 1108, 1059, 742. MS (CI, m/z, %): 288 (M^+^+1, 100), 146 (25). MS (EI, m/z, %): 287 (M^+^, 13), 146 (100). HRMS: Calcd. for C_20_H_33_N: 287.2613. Found: 287.2614.

### 1-Dodecyl-1,2,3,4-tetrahydroquinoline (2d)

The compound was prepared according to General Procedure I from 0.514 g (3.86 mmol) 1,2,3,4-tetrahydroquinoline and 2.218 g (7.49 mmol) 1-iodododecane to give 0.90 g (78%) of **2d** as a pale yellow oil.

Alternative approach: 0.594 g (1.89 mmol) *N*-dodecanoyl-1,2,3,4-tetrahydroquinoline (**6**) was dissolved in 30 mL of dry THF and 270 mg (3.8 mmol) of LiAlH_4_ were added. The suspension was refluxed for 5 h, then quenched with 30 mL aqueous 10% NaOH and extracted with ethyl acetate (3 × 30 mL). The combined organic layers were dried over Na_2_SO_4_, the solvent was evaporated, and the residue was purified by flash column chromatography to give 0.503 g (88%) of **2d** as a pale yellow oil. δ 0.88 (t, *J* = 7.2 Hz, 3 H, CH_3_), 1.26–1.31 (m, 18 H, 9 CH_2_), 1.57 (m, 2 H, CH_2_), 1.93 (tt, *J* = 5.7 Hz, *J* = 6.4 Hz, 2 H, CH_2,_ 3-H), 2.74 (t, *J* = 6.4 Hz, 2 H, CH_2_, 4-H), 3.21 (t, *J* = 7.6 Hz, 2 H, CH_2_), 3.26 (t, *J* = 5.7 Hz, 2 H, CH_2_, 2-H), 6.53 (dd, *J* = 7.8 Hz, *J* = 7.9 Hz, 1 H, 6-H), 6.55 (d, *J* = 7.8 Hz, 1 H, 8-H), 6.93 (d, *J* = 7.9 Hz, 1 H, 5-H), 7.03 (dd, *J* = 7.8 Hz, *J* = 7.8 Hz, 1 H, 7-H). ^13^C-NMR (100 MHz, CDCl_3_, TMS): δ 14.2 (CH_3_), 22.2 (CH_2_), 22.7 (CH_2_), 26.1 (CH_2_), 27.3 (CH_2_), 28.2 (CH_2_), 29.4 (CH_2_), 29.6 (CH_2_), 29.6 (CH_2_), 29.6 (CH_2_), 29.7(CH_2_), 29.7 (CH_2_), 31.9 (CH_2_), 49.4 (CH_2_), 51.5 (CH_2_), 110.4 (arom. CH), 115.1 (arom. CH), 122.1 (quat. arom. C), 127.0 (arom. CH), 129.1 (arom. CH), 145.3 (quat. C). IR (KBr), ν, cm^−1^: 3407, 3065, 3020, 2923, 2852, 1660, 1602, 1504, 1456, 1346, 1195, 1109, 1059, 742. MS (CI, m/z, %): 302 (M^+^+1, 100), 146 (31). MS (EI, m/z, %): 301 (M^+^, 9), 146 (100). HR-MS: Calcd. for C_21_H_35_N: 301.2769. Found: 301.2770.

### 1-Nonyl-1,2,3,4-tetrahydroquinoline hydrochloride (3a)

The compound was prepared according to General Procedure II from 0.700 g (2.70 mmol) 1-nonyl-1,2,3,4-tetrahydroquinoline (**2a**) to give 0.48 g (60%) of **3a** as a yellow-orange solid. M.p.: 65°C (diethyl ether). ^1^H-NMR (400 MHz, CDCl_3_, TMS): δ 0.87 (t, *J* = 7.2 Hz, 3 H, CH_3_), 1.18–1.39 (m, 12 H, 6 CH_2_), 1.91 (m, 2 H, CH_2_), 2.22 (tt, *J* = 5.9 Hz, *J* = 6.9 Hz, 2 H, CH_2_, 3-H), 2.97 (t, *J* = 6.9 Hz, 2 H, CH_2_, 4-H), 3.35 (t, *J* = 8.5 Hz, 2 H, CH_2_), 3.59 (t, *J* = 5.9 Hz, 2 H, CH_2_, 2-H), 7.23 (m, 1 H, arom. CH), 7.34 (m, 2 H, 2 arom. CH), 7.69 (m, 1 H, arom. CH), 14.0 (s, 1 H, NH). ^13^C-NMR (100 MHz, CDCl_3_, TMS): δ 14.1 (CH_3_), 16.3 (CH_2_), 22.6 (CH_2_), 24.4 (CH_2_), 24.8 (CH_2_), 26.7 (CH_2_), 29.0 (CH_2_), 29.11 (CH_2_), 29.35 (CH_2_), 31.7 (CH_2_), 48.0 (CH_2_), 58.7 (CH_2_), 124.2 (arom. CH), 127.9 (arom. CH), 129.4 (arom. CH), 130.5 (quat. C), 130.6 (arom. CH), 136.0 (quat. C). IR (KBr), ν, cm^−1^: 3064, 2930, 2855, 2437, 2254, 1591, 1501, 1472, 1171, 1066, 760. MS (CI, m/z, %): 260 (M^+^-Cl, 100), 146 (19). MS (EI, m/z, %): 259 (M^+^-HCl, 12), 146 (100). HR-MS: Calcd. for C_18_H_29_N: 259.2300. Found: 259.2306.

### 1-Decyl-1,2,3,4-tetrahydroquinoline hydrochloride (3b)

The compound was prepared according to General Procedure II from 0.790 g (2.89 mmol) 1-decyl-1,2,3,4-tetrahydroquinoline (**2b**) to give 0.43 g (49%) of **3b** as a yellow-orange solid. M.p.: 61°C (diethyl ether). ^1^H-NMR (400 MHz, CDCl_3_, TMS): δ 0.87 (t, *J* = 7.2 Hz, 3 H, CH_3_), 1.24–1.31 (m, 14 H, 7 CH_2_), 1.91 (m, 2 H, CH_2_), 2.21 (tt, *J* = 5.8 Hz, *J* = 6.9 Hz, 2 H, CH_2_, 3 -H), 2.96 (t, *J* = 6.9 Hz, 2 H, CH_2_, 2-H), 3.34 (t, *J* = 8.5 Hz, 2 H, CH_2_), 3.58 (t, *J* = 5.8 Hz, 2 H, CH_2_), 7.22 (m, 1 H, arom. CH), 7.34 (m, 2 H, 2 arom. CH), 7.69 (m, 1 H, arom. CH), 14.06 (s, 1 H, NH). ^13^C-NMR (100 MHz, CDCl_3_, TMS): δ 14.1 (CH_3_), 16.3 (CH_2_), 22.7 (CH_2_), 24.4 (CH_2_), 24.8 (CH_2_), 26.8 (CH_2_), 29.0 (CH_2_), 29.2 (CH_2_), 29.4 (2 CH_2_), 31.8 (CH_2_), 48.0 (CH_2_), 58.7 (CH_2_), 124.2 (arom. CH), 127.9 (arom. CH), 129.4 (arom. CH), 130.4 (quat. C), 130.6 (arom. CH), 136.0 (quat. C). IR (KBr), ν, cm^−1^: 3062, 2922, 2853, 2438, 2260, 1592, 1501, 1471, 1170, 1067, 759. MS (CI, m/z, %): 274 (M^+^-Cl, 100), 146 (28). MS (EI, m/z, %): 273 (M^+^-HCl, 11), 146 (100). HR-MS: Calcd. for C_19_H_31_N: 273.2456. Found: 273.2458.

### 1-Undecyl-1,2,3,4-tetrahydroquinoline hydrochloride (3c)

The compound was prepared according to General Procedure II from 0.763 g (2.66 mmol) 1-undecyl-1,2,3,4-tetrahydroquinoline (**2c**) to give 0.37 g (43%) of **3c** as a yellow-orange solid. M.p.: 52°C (diethyl ether). ^1^H-NMR (400 MHz, CDCl_3_, TMS): δ 0.88 (t, *J* = 7.2 Hz, 3 H, CH_3_), 1.24–1.31 (m, 16 H, 8 CH_2_), 1.85 (m, 2 H, CH_2_), 2.22 (m, 2 H, CH_2_, 3-H), 2.97 (t, *J* = 6.9 Hz, 2 H, CH_2_, 4-H), 3.35 (t, *J* = 8.5 Hz, 2 H, CH_2_), 3.59 (m, 2 H, CH_2_, 2-H), 7.23 (m, 1 H, arom. CH), 7.34 (m, 2 H, 2 arom. CH), 7.69 (m, 1 H, arom. CH), 14.05 (s, 1 H, NH). ^13^C-NMR (100 MHz, CDCl_3_, TMS): δ 14.1 (CH_3_), 16.3 (CH_2_), 22.7 (CH_2_), 24.4 (CH_2_), 24.8 (CH_2_), 26.8 (CH_2_), 29.0 (CH_2_), 29.3 (CH_2_), 29.4 (CH_2_), 29.5 (CH_2_), 29.5 (CH_2_), 31.9 (CH_2_), 48.0 (CH_2_), 58.7 (CH_2_), 124.2 (arom. CH), 127.9 (arom. CH), 129.4 (arom. CH), 130.5 (quat. C), 130.6 (arom. CH), 136.0 (quat. C). IR (KBr), ν, cm^−1^: 3062, 2921, 2852, 2442, 2261, 1597, 1500, 1470, 1069, 759. MS (CI, m/z, %): 288 (M^+^-Cl, 100), 146 (33). MS (EI, m/z, %): 287 (M^+^-HCl, 10), 146 (100). HR-MS: Calcd. for C_20_H_33_N: 287.2613. Found: 287.2650.

### 1-Dodecyl-1,2,3,4-tetrahydroquinoline hydrochloride (3d)

The compound was prepared according to General Procedure II from 0.788 g (2.62 mmol) 1-dodecyl-1,2,3,4-tetrahydroquinoline (**2d**) to give 0.47 g (53%) of **3d** as a brown solid. M.p.: 49°C (diethyl ether). ^1^H-NMR (400 MHz, CDCl_3_, TMS): δ 0.88 (t, *J* = 7.2 Hz, 3 H, CH_3_), 1.25–1.31 (m, 18 H, 9 CH_2_), 1.77 (m, 2 H, CH_2_), 2.12 (m, 2 H, CH_2_, 3-H), 2.87 (t, *J* = 6.3 Hz, 2 H, CH_2_, 4-H), 3.29 (t, *J* = 8.5 Hz, 2 H, CH_2_), 3.46 (t, *J* = 6.1 Hz, 2 H, CH_2_, 2-H), 7.23 (m, 1 H, arom. CH), 7.33 (m, 2 H, 2 arom. CH), 7.70 (m, 1 H, arom. CH), 14.14 (s, 1 H, NH). ^13^C-NMR (100 MHz, CDCl_3_, TMS): δ 14.14 (CH_3_), 16.3 (CH_2_), 22.7 (CH_2_), 24.4 (CH_2_), 24.8 (CH_2_), 26.8 (CH_2_), 29.1 (CH_2_), 29.3 (CH_2_), 29.3 (CH_2_), 29.5 (CH_2_), 29.6 (2 CH_2_), 31.9 (CH_2_), 48.0 (CH_2_), 58.7 (CH_2_), 124.2 (arom. CH), 127.9 (arom. CH), 129.4 (arom. CH), 130.5 (quat. C), 130.6 (arom. CH), 136.0 (quat. C). IR (KBr), ν, cm^−1^: 3419, 3064, 2924, 2853, 2336, 1601, 1504, 1458, 1346, 1195, 1110, 1061, 742. MS (CI, m/z, %): 302 (M^+^-Cl, 100), 146 (37). MS (EI, m/z, %): 301 (M^+^-HCl, 10), 146 (100). HR-MS: Calcd. for C_21_H_35_N: 301.2769. Found: 301.2742.

### (±)-trans-1-Nonyldecahydroquinoline (4a)

The compound was prepared according to General Procedure I from 0.505 g (3.63 mmol) (±)-*trans*-decahydroquinoline and 1.025 g (4.95 mmol) 1-bromononane to give 0.62 g (64%) of **4a** as a pale yellow oil. ^1^H-NMR (400 MHz, CDCl_3_, TMS): δ 0.88 (t, *J* = 7.2 Hz, 3 H, CH_3_), 0.94 (m, 1 H, CH_2_), 0.98 (m, 1 H, CH_2_), 1.09 (m, 1 H, CH_2_), 1.19 (m, 1 H, CH_2_), 1.21 (m, 1 H, CH), 1.23 (m, 1 H, CH_2_), 1.26 (m, 12 H, 6 CH_2_), 1.30 (m, 1 H, CH_2_), 1.41 (m, 1 H, CH_2_), 1.56 (m, 1 H, CH_2_), 1.60 (m, 1 H, CH_2_), 1.62 (m, 2 H, CH_2_), 1.65 (m, 1H, CH_2_), 1.75 (m, 1 H, CH), 1.78 (m, 1 H, CH_2_), 2.06 (m, 1 H, CH_2_), 2.20 (m, 1 H, CH_2_), 2.45 (m, 1 H, CH_2_), 2.65 (m, 1 H, CH_2_), 2.92 (m, 1 H, CH_2_). ^13^C-NMR (100 MHz, CDCl_3_, TMS): δ 14.2 (CH_3_), 22.8 (CH_2_), 24.1 (CH_2_), 25.9 (CH_2_), 25.9 (CH_2_), 26.1 (CH_2_), 27.9 (CH_2_), 29.4 (CH_2_), 29.7 (CH_2_), 29.7 (CH_2_), 30.2 (CH_2_), 32.0 (CH_2_), 32.7 (CH_2_), 33.3 (CH_2_), 42.0 (CH), 53.2 (CH_2_), 53.6 (CH_2_), 65.8 (CH). IR (KBr), ν, cm^−1^: 2924, 2854, 1459, 1448, 1378, 1366, 1239, 1095. MS (CI, m/z, %): 266 (M^+^+1, 100). MS (EI, m/z, %): 265 (M^+^, 6), 264 (3), 222 (49), 152 (100). HR-MS: Calcd. for C_18_H_35_N: 265.2769. Found: 265.2748.

### (±)-trans-1-Decyldecahydroquinoline (4b)

The compound was prepared according to General Procedure I from 0.512 g (3.68 mmol) (±)-*trans*-decahydroquinoline and 1.104 g (4.99 mmol) 1-bromodecane to give 0.46 g (45%) of **4b** as a pale yellow oil. ^1^H-NMR (400 MHz, CDCl_3_, TMS): δ 0.88 (t, *J* = 7.2 Hz, 3 H, CH_3_), 0.96 (m, 1 H, CH_2_), 0.98 (m, 1 H, CH_2_), 1.09 (m, 1 H, CH_2_), 1.21 (m, 4 H, 2 CH_2_), 1.23 (m, 1 H, CH), 1.26 (m, 14 H, 7 CH_2_), 1.41 (m, 1 H, CH_2_), 1.56 (m, 1 H, CH_2_), 1.58 (m, 1 H, CH_2_), 1.61 (m, 2 H, CH_2_), 1.73 (m, 1 H, CH_2_), 1.77 (m, 1 H, CH), 2.06 (m, 1 H, CH_2_), 2.20 (m, 1H, CH_2_), 2.45 (m, 1 H, CH_2_), 2.65 (m, 1 H, CH_2_), 2.92 (m, 1 H, CH_2_). ^13^C-NMR (100 MHz, CDCl_3_, TMS): δ 14.2 (CH_3_), 22.8 (CH_2_), 24.1 (CH_2_), 25.8 (CH_2_), 25.9 (CH_2_), 26.0 (CH_2_), 27.9 (CH_2_), 29.4 (CH_2_), 29.7 (CH_2_), 29.7 (CH_2_), 29.7 (CH_2_), 30.1 (CH_2_), 32.0 (CH_2_), 32.7 (CH_2_), 33.3 (CH_2_), 41.9 (CH), 53.1 (CH_2_), 53.5 (CH_2_), 65.8 (CH). IR (film), ν, cm^−1^: 2924, 2853, 1460, 1447, 1378, 1366, 1239, 1095. MS (CI, m/z, %): 280 (M+1^+^, 100). MS (EI, m/z, %): 279 (M^+^, 2), 237 (4), 236 (23), 152 (100). HR-MS: Calcd. for C_19_H_37_N: 279.2926. Found: 279.2901.

### (±)-trans-1-Undecyldecahydroquinoline (4c)

The compound was prepared according to General Procedure I from 0.512 g (3.68 mmol) (±)-*trans*-decahydroquinoline and 1.211 g (5.14 mmol) 1-bromoundecane to give 0.44 g (41%) of **4c** as a pale yellow oil. ^1^H-NMR (400 MHz, CDCl_3_, TMS): δ 0.88 (t, *J* = 7.2 Hz, 3 H, CH_3_), 0.95 (m, 1 H, CH_2_), 0.98 (m, 1 H, CH_2_), 1.09 (m, 1 H, CH_2_), 1.21 (m, 2 H, CH_2_), 1.23 (m, 1 H, CH), 1.26 (m, 18H, 9 CH_2_), 1.42 (m, 1 H, CH_2_), 1.58 (m, 2 H, CH_2_), 1.61 (m, 2 H, CH_2_), 1.73 (m, 1 H, CH_2_), 1.77 (m, 1 H, CH), 2.05 (m, 1 H, CH_2_), 2.20 (m, 1 H, CH_2_), 2.46 (m, 1 H, CH_2_), 2.64 (m, 1 H, CH_2_), 2.92 (m, 1 H, CH_2_). ^13^C-NMR (100 MHz, CDCl_3_, TMS): δ 14.2 (CH_3_), 22.8 (CH_2_), 24.0 (CH_2_), 25.8 (CH_2_), 25.9 (CH_2_), 26.0 (CH_2_), 27.9 (CH_2_), 29.4 (CH_2_), 29.6 (CH_2_), 29.7 (CH_2_), 29.7 (CH_2_), 29.7 (CH_2_), 30.1 (CH_2_), 32.0 (CH_2_), 32. 7 (CH_2_), 33.3 (CH_2_), 41.9 (CH), 53.1 (CH_2_), 53.5 (CH_2_), 65.8 (CH). IR (film), ν, cm^−1^: 2924, 2853, 1460, 1448, 1378, 1366, 1239, 1094. MS (CI, m/z, %): 294 (M^+^+1, 100). MS (EI, m/z, %): 293 (M^+^, 2), 250 (23), 152 (100). HR-MS: Calcd. for C_20_H_39_N: 293.3082. Found: 293.3081.

### (±)-trans-1-Dodecyldecahydroquinoline (4d)

The compound was prepared according to General Procedure I from 0.535 g (3.84 mmol) (±)-*trans*-decahydroquinoline and 1.485 g (5.01 mmol)1-iodododecane and to give 1.08 g (3.53 mmol) (92%) of **4d** as a pale yellow oil. ^1^H-NMR (400 MHz, CDCl_3_, TMS): δ 0.88 (t, *J* = 7.2 Hz, 3 H, CH_3_), 0.95 (m, 1 H, CH_2_), 0.97 (m, 1 H, CH_2_), 1.06 (m, 1 H, CH_2_), 1.20 (m, 1 H, CH_2_), 1.21 (m, 1 H, CH), 1.26 (m, 20 H, 10 CH_2_), 1.41 (m, 1 H, CH_2_), 1.56 (m, 1 H, CH_2_), 1.60 (m, 1 H, CH_2_), 1.62 (m, 2 H, CH_2_), 1.73 (m, 1 H, CH), 1.77 (m, 1 H, CH_2_), 2.05 (m, 1 H, CH_2_), 2.17 (m, 1 H, CH_2_), 2.20 (m, 1 H, CH_2_), 2.44 (m, 1 H, CH_2_), 2.65 (m, 1 H, CH_2_), 2.91 (m, 1 H, CH_2_). ^13^C-NMR (100 MHz, CDCl_3_, TMS): δ 14.22 (CH_3_), 22.78 (CH_2_), 24.1 (CH_2_), 25.9 (CH_2_), 25.9 (CH_2_), 26.0 (CH_2_), 27.9 (CH_2_), 29.4 (CH_2_), 29.7 (CH_2_), 29.7 (CH_2_), 29.7 (CH_2_), 29.8 (CH_2_), 30.1 (CH_2_), 31.0 (CH_2_), 32.0 (CH_2_), 32.7 (CH_2_), 33.3 (CH_2_), 42.0 (CH), 53.1 (CH_2_), 53.6 (CH_2_), 65.8 (CH). IR (KBr), ν, cm^−1^: 2923, 2853, 1460, 1448, 1378, 1366, 1239, 1095. MS (CI, m/z, %): 308 (M^+^+1, 95), 152 (100). MS (EI): m/z (%) = 307 (M^+^, 2), 264 (24), 152 (100). HR-MS: Calcd. for C_21_H_41_N: 307.3239. Found: 307.3251.

### (±)-trans-1-Nonyldecahydroquinoline hydrochloride (5a)

The compound was prepared according to General Procedure II from 0.380 g (1.43 mmol) (±)-*trans*-1-nonyldecahydroquinoline (**4a**) of to give 0.39 g (91%) of **5a** as a white solid. M.p.: 130°C (diethyl ether). ^1^H-NMR (400 MHz, CD_2_Cl_2_, TMS): δ 0.88 (t, *J* = 7.2 Hz, 3H, CH_3_), 1.01 (m, 1 H, CH_2_), 1.04 (m, 1 H, CH_2_), 1.14 (m, 1 H, CH_2_), 1.28 (m, 10 H, 5 CH_2_), 1.36 (m, 2 H, CH_2_), 1.58 (m, 1 H, CH_2_), 1.65 (m, 1 H, CH_2_), 1.71 (m, 1 H, CH_2_), 1.76 (m, 2 H, CH_2_), 1.78 (m, 2 H, CH_2_), 1.80 (m, 1 H, CH_2_), 1.92 (m, 1 H, CH_2_), 2.00 (m, 1 H, CH), 2.05 (m, 1 H, CH_2_), 2.31 (m, 1 H, CH_2_), 2.51 (m, 1 H, CH), 2.78 (m, 1 H, CH_2_), 2.99 (m, 2 H, CH_2_), 3.38 (m, 1 H, CH_2_). ^13^C-NMR: (100 MHz, CD_2_Cl_2_, TMS): δ 14.3 (CH_3_), 21.9 (CH_2_), 23.1 (CH_2_), 23.1 (CH_2_), 25.0 (CH_2_), 25.7 (CH_2_), 27.3 (CH_2_), 27.4 (CH_2_), 29.5 (CH_2_), 29.6 (CH_2_), 29.8 (CH_2_), 30.9 (CH_2_), 32.2 (CH_2_), 33.2 (CH_2_), 39.4 (CH), 52.2 (CH_2_), 53.3 (CH_2_), 66.9 (CH). IR (KBr), ν, cm^−1^: 2924, 2858, 2516, 2492, 1468, 1452, 1376, 1084, 1047. MS (CI, m/z, %): 266 (M^+^-Cl, 100), 152 (45). MS (EI): m/z (%) = 265 (M^+^-HCl, 2), 222 (25), 152 (100). HR-MS: Calcd. for C_18_H_36_N: 266.2848. Found: 266.2846.

### (±)-trans-1-Decyldecahydroquinoline hydrochloride (5b)

The compound was prepared according to General Procedure II from 0.214 g (0.77 mmol) (±)-*trans*-1-decyldecahydroquinoline (**4b**) to give 0.21 g (86%) of **5b** as a white solid. M.p.: 128°C (diethyl ether). ^1^H-NMR (400 MHz, CDCl_3_, TMS): δ 0.88 (t, *J* = 7.2 Hz, 3 H, CH_3_), 1.01 (m, 1 H, CH_2_), 1.14 (m, 1 H, CH_2_), 1.28 (m, 14 H, 7 CH_2_), 1.34 (m, 2 H, CH_2_), 1.58 (m, 1 H, CH_2_), 1.65 (m, 1 H, CH_2_), 1.75 (m, 2 H, CH_2_), 1.77 (m, 2 H, CH_2_), 1.80 (m, 1 H, CH_2_), 1.91 (m, 1 H, CH_2_), 2.00 (m, 1H, CH), 2.05 (m, 1H, CH_2_), 2.30 (m, 1H, CH_2_), 2.50 (m, 1 H, CH), 2.79 (m, 1 H, CH_2_), 3.00 (m, 2 H, CH_2_), 3.37 (m, 1 H, CH_2_). ^13^C-NMR: (100 MHz, CD_2_Cl_2_, TMS): δ 14.3 (CH_3_), 21.9 (CH_2_), 23.1 (CH_2_), 23.1 (CH_2_), 25.0 (CH_2_), 25.7 (CH_2_), 27.3 (CH_2_), 27.4 (CH_2_), 29.5 (CH_2_), 29.7 (CH_2_), 29.8 (CH_2_), 29.9 (CH_2_), 30.8 (CH_2_), 32.3 (CH_2_), 33.2 (CH_2_), 39.4 (CH), 52.2 (CH_2_), 53.3 (CH_2_), 66.9 (CH). IR (KBr), ν, cm^−1^: 2924, 2855, 2520, 2493, 1464, 1453, 1377, 1072, 1046. MS (CI, m/z, %): 280 (M^+^-Cl, 100), 152 (49). MS (EI): m/z (%) = 279 (M^+^-HCl, 2), 236 (24), 152 (100). HR-MS: Calcd. for C_19_H_38_N: 280.3004. Found: 280.3000.

### (±)-trans-1-Undecyldecahydroquinoline hydrochloride (5c)

The compound was prepared according to General Procedure II from 0.194 g (0.66 mmol) (±)-*trans*-1-undecyldecahydroquinoline (**4c**) to give 0.18 g (83%) of **5c** as a white solid. M.p.: 119°C (diethyl ether). ^1^H-NMR (400 MHz, CDCl_3_, TMS): δ 0.88 (t, *J* = 7.2 Hz, 3 H, CH_3_), 1.02 (m, 1 H, CH_2_), 1.14 (m, 1 H, CH_2_), 1.27 (m, 16 H, 8 CH_2_), 1.33 (m, 2 H, CH_2_), 1.56 (m, 1 H, CH_2_), 1.65 (m, 1 H, CH_2_), 1.76 (m, 2 H, CH_2_), 1.78 (m, 2 H, CH_2_), 1.80 (m, 1 H, CH_2_), 1.91 (m, 1 H, CH_2_), 2.00 (m, 1 H, CH), 2.04 (m, 1 H, CH_2_), 2.31 (m, 1 H, CH_2_), 2.50 (m, 1 H, CH), 2.78 (m, 1 H, CH_2_), 3.01 (m, 2 H, CH_2_), 3.36 (m, 1 H, CH_2_). ^13^C-NMR: (100 MHz, CD_2_Cl_2_, TMS): δ 14.3 (CH_3_), 21.9 (CH_2_), 23.1 (CH_2_), 23.1 (CH_2_), 25.0 (CH_2_), 25.7 (CH_2_), 27.3 (CH_2_), 27.4 (CH_2_), 29.5 (CH_2_), 29.7 (CH_2_), 29.8 (CH_2_), 29.9 (CH_2_), 30.0 (CH_2_), 30.8 (CH_2_), 32.3 (CH_2_), 33.17 (CH_2_), 39.4 (CH), 52.2 (CH_2_), 53.3 (CH_2_), 66.9 (CH). IR (KBr), ν, cm^−1^: 2923, 2855, 2519, 2493, 1469, 1454, 1376, 1074, 1046. MS (CI, m/z, %): 294 (M^+^-Cl, 100), 152 (57). MS (EI): m/z (%) = 293 (M^+^-HCl, 2), 250 (16), 152 (100). HR-MS: Calcd. for C_20_H_40_N: 294.3161. Found: 294.3164.

### (±)-trans-1-Dodecyldecahydroquinoline hydrochloride (5d)

The compound was prepared according to General Procedure II from 0.796 g (2.59 mmol) (±)-*trans*-1-dodecyldecahydroquinoline (**4d**) to give 0.76 g (2.20 mmol) (85%) of **5d** as a white solid. M.p.: 113°C (diethyl ether). ^1^H-NMR (400 MHz, CDCl_3_, TMS): δ 0.88 (t, *J* = 7.2 Hz, 3 H, CH_3_), 1.01 (m, 1 H, CH_2_), 1.13 (m, 1 H, CH_2_), 1.27 (m, 18 H, 9 CH_2_), 1.32 (m, 2 H, CH_2_), 1.56 (m, 1 H, CH_2_), 1.65 (m, 1 H, CH_2_), 1.76 (m, 2 H, CH_2_), 1.79 (m, 2 H, CH_2_), 1.80 (m, 1 H, CH_2_), 1.91 (m, 1 H, CH_2_), 2.00 (m, 1 H, CH), 2.05 (m, 1 H, CH_2_), 2.28 (m, 1 H, CH_2_), 2.50 (m, 1 H, CH), 2.79 (m, 1 H, CH_2_), 3.03 (m, 2 H, CH_2_), 3.37 (m, 1 H, CH_2_). ^13^C-NMR: (100 MHz, CD_2_Cl_2_, TMS): δ 14.3 (CH_3_), 22.0 (CH_2_), 23.1 (CH_2_), 23.1 (CH_2_), 25.0 (CH_2_), 25.7 (CH_2_), 27.2 (CH_2_), 27.4 (CH_2_), 29.5 (CH_2_), 29.8 (CH_2_), 29.8 (CH_2_), 29.9 (CH_2_), 30.0 (CH_2_), 30.0 (CH_2_), 30.8 (CH_2_), 32.3 (CH_2_), 33.2 (CH_2_), 39. 5 (CH), 52.3 (CH_2_), 53.4 (CH_2_), 67.0 (CH). IR (KBr), ν, cm^−1^: 2923, 2852, 2520, 2494, 1466, 1453, 1393, 1075, 1046. MS (CI): m/z (%) = 308 (M^+^-Cl, 100), 152 (68). MS (EI): m/z (%) = 307 (M^+^-HCl, 2), 264 (20), 152 (100). HR-MS: Calcd. C_21_H_42_N: 308.3317. Found: 308.3315.

### N-Dodecanoyl-1,2,3,4-tetrahydroquinoline (6)

For this compound’s preparation, 0.514 g (3.86 mmol) 1,2,3,4-tetrahydroquinoline and 1.232 g dodecanoyl chloride (5.63 mmol) were dissolved in 30 mL of dry 1,2-dimethoxyethane. Five mL of dimethyl ethyl amine were added and the mixture was stirred for 10 h. The solvent was evaporated, the residue was dissolved in 30 mL aqueous 10% NaOH, and extracted with ethyl acetate (3 × 30 mL). The combined organic layers were dried over Na_2_SO_4_, the solvent was evaporated, and the residue was purified by flash column chromatography to give 0.88 g (72%) of **6** as a pale yellow solid. M.p.: 37°C (isohexane/ethyl acetate). ^1^H-NMR (400 MHz, CDCl_3_, TMS): δ 0.88 (t, *J* = 7.2 Hz, 3 H, CH_3_), 1.23 (m, 16 H, 8 CH_2_), 1.65 (m, 2 H, CH_2_), 1.96 (tt, *J* = 6.6 Hz, *J* = 6.6 Hz, 2 H, CH_2_, 3-H), 2.49 (t, *J* = 7.6 Hz, 2 H, CH_2_), 2.71 (t, *J* = 6.6 Hz, 2 H, CH_2_, 4-H), 3.79 (t, *J* = 6.6 Hz, 2 H, CH_2,_ 2-H), 7.15 (m, 4 H, 4 arom. CH). ^13^C-NMR (100 MHz, CDCl_3_, TMS): δ 14.1 (CH_3_), 22.7 (CH_2_), 24.2 (CH_2_), 25.9 (CH_2_), 26.8 (CH_2_), 29.3 (CH_2_), 29.3 (CH_2_), 29.4 (CH_2_), 29.5 (CH_2_), 29.6 (CH_2_), 29.6 (CH_2_), 31.9 (CH_2_), 34.5 (CH_2_), 42.8 (CH_2_), 124.7 (quat. C), 125.1 (arom. CH), 126.0 (arom. CH), 128.4 (2 arom. CH), 139.3 (quat. C), 173.2 (quat. C). IR (KBr), ν, cm^−1^: 3273, 3056, 2921, 2849, 1655, 1605, 1579, 1493, 1463, 1388, 1244, 1169, 1111, 1069, 756. MS (CI, m/z, %): 316 (M^+^+1, 100), 133 (47). MS (EI, m/z, %): 315 (M^+^, 4), 133 (100). HR-MS: Calcd. for C_21_H_33_NO: 315.2562. Found: 315.2561.

## Authors’ Statement

The authors declare no conflict of interest.
